# The Effect of Maturity and Extraction Solvents on Bioactive Compounds and Antioxidant Activity of Mulberry (*Morus alba*) Fruits and Leaves

**DOI:** 10.3390/molecules27082406

**Published:** 2022-04-08

**Authors:** Centhyea Chen, Ruzaidi Azli Mohd Mokhtar, Muhamad Shirwan Abdullah Sani, Nor Qhairul Izzreen Mohd Noor

**Affiliations:** 1 Faculty of Food Science and Nutrition, Universiti Malaysia Sabah, Kota Kinabalu 88400, Malaysia; centhyea97@gmail.com; 2 Biotechnology Research Institute, Universiti Malaysia Sabah, Kota Kinabalu 88400, Malaysia; ruzaidi@ums.edu.my; 3 International Institute for Halal Research and Training, Level 3, KICT Building, International Islamic University Malaysia, Jalan Gombak, Kuala Lumpur 53100, Malaysia; shirwansany@iium.edu.my; 4 Konsortium Institut Halal IPT Malaysia, Ministry of Higher Education, Block E8, Complex E, Federal Government Administrative Centre, Putrajaya 62604, Malaysia

**Keywords:** bioactive compounds, antioxidant activity, UHPLC-DAD, chlorogenic acid, rutin, *Morus alba* Linnaeus, mulberry fruits, mulberry leaves, principal component analysis (PCA)

## Abstract

Cultivation location, maturity levels, and extraction solvents could affect the bioactive compounds and biological activities of mulberry (*Morus alba* Linnaeus). The lack of study on Malaysia-grown mulberry causes its underutilization. This study investigated the bioactive compound content and the antioxidant activity of Sabah-grown mulberry at two different maturity stages (fruits: red mature and black fully ripe; leaves: young and mature) extracted using 70% (*v*/*v*) methanol, 60% (*v*/*v*) ethanol, and 65% (*v*/*v*) acetone. Analyses showed that mulberry fruits demonstrated maturity-dependent increment (except UHPLC-DAD quantification), while the leaves revealed maturity-dependent reduction. Principal component analysis (PCA) displayed 65% (*v*/*v*) acetone black fully ripe fruits as the best phenolics and antioxidant sources. However, the 60% (*v*/*v*) ethanol black fully ripe fruits contained 20.08–68.43% higher total anthocyanins. Meanwhile, the 65% (*v*/*v*) acetone and 70% (*v*/*v*) methanol red mature fruits were higher in chlorogenic acid (27.53–47.12%) and rutin (31.42–35.92%) than other fruit extracts, respectively. For leaves, 65% (*v*/*v*) acetone young leaves were the best phenolics and antioxidant sources. However, the 60% (*v*/*v*) ethanol young leaves possessed greater chlorogenic acid (19.56–74.11%) than other leaf extracts. Overall, Malaysia-grown mulberry is rich in phenolics and antioxidants, suggesting its potential application in food and pharmaceutical products.

## 1. Introduction

Free radicals are the natural by-product produced during body cellular metabolism that are significant in homeostasis, gene expression, cell signaling, ion transportation, and the apoptosis process [1]. However, the excess presence of free radicals holds fatal damage to cell components and structures, triggering the oxidative stress-induced pathogenesis of various diseases, including cataracts, inflammatory disease, diabetes, autism, cardiovascular disease, cancer, and neurodegenerative diseases [1]. Therefore, exogenous intake of antioxidants is needed to boost the body antioxidant level, amend oxidative stress-instigated damage, and inhibit oxidative chain reaction [2].

White mulberry (*Morus alba* Linnaeus) is a China-originated plant that is classified under the genus *Morus* L. from the Moraceae family [3]. This plant has been traditionally utilized for its therapeutic effects, and the medicinal properties of mulberry fruits and leaves have been well recorded in *Chinese Pharmacopoeia* and *British Herbal Pharmacopoeia* [4,5]. Mulberry contains high nutraceutical values based on its low fat content (2–3.5%) [6] but high levels of carbohydrate (9–71%), protein (13–34.2%), fiber (5.4–38.4%) [6,7,8], and organic acids (5.60 mg/g fresh weight) [9]. Moreover, the 42–44% total amino acid obtained from mulberry in Jiang and Nie [9] was equivalent to the content in fish (40.7%) and milk (44%), making it a good protein source. Mulberry also contained abundant macro elements, in which the contents of zinc (50.50 mg/100 g) [10] and iron (11.2–27.6 mg/g) [11] were higher than those in iron-rich sesame soy and shrimp paste (9.4–11.6 mg/100 g) [9]. The high contents of micronutrients and macronutrients in mulberry show its great potentiality in fulfilling the recommended nutrient intakes (RNIs) for Malaysians [12] and the dietary reference intakes (DRIs) for Americans [13].

Their rich amount of biomolecules and antioxidative bioactive compounds, such as phenolic acids, flavonoids, flavonols, anthocyanins, and others, have been extensively described in multiple reports [8,14,15]. In Tunisian mulberry, Jelled et al. [15] obtained 46.01–54.29 mg GAE/g extract of total phenolic content (TPC), 30.31–43.91 mg CE/g extract of total flavonoid content (TFC), 9.46–14.2 mg CE/g extract of total tannin content, and 0.83–2.44 µg/g DW of total anthocyanin content (TAC). The study also expressed mulberry’s potent antioxidant activity through 2.2-dyphenyl-1-pikrilhidrazil (DPPH), radical cation 2,2′-azino-bis(3-ethylbenzthiazoline-6-sulphonic acid) (ABTS), and ferric reduction antioxidant power (FRAP) assays (IC_50_ of 1.13–3.97 mg/mL, 2.58–3.73 mg/mL, and 0.52–0.65 mg/mL, respectively) [15]. Meanwhile, mulberry leaves in Yu et al. [16] expressed 8.76–20.26 mg/g DW of TPC, 21.36–56.41 mg/g DW of TFC, 2.56–10.24 mg/g DW of chlorogenic acid content, and 0.42–4.31 mg/g DW of rutin content. The leaves also exhibited high antioxidant values in DPPH (33.22–56.37 µmol TE/g DW), ABTS (51.28–69.13 µmol TE/g DW), and FRAP (91.62–149.15 µmol AAE/g DW). Aside from being antioxidative, the compound richness of mulberry is also the main contributor for its various pharmacological effects, including anti-inflammatory, anticancer, antidiabetic, antihyperlipidemic, antiobesity, antihypertension, antimicrobial, antiviral, and neuroprotective effects [8,17,18]. Owing to its beneficial properties and desirable flavor, mulberry fruits are mostly integrated in the food industry, while the leaves are increasingly relished as a tea for its rich γ-aminobutyric acid content [19]. Moreover, several studies have successfully reported their usage of mulberry as food antioxidant, colorants, forticant, flavorant, preservative, and antimicrobial agent [8,20,21]. Hence, indicating the increasing fame and functionality of mulberry as food ingredients.

The high adaptability of this mulberry plant to various topographies leads to their wide cultivation in various-season countries [3]. This includes Malaysia with 3 hectares of mulberry plantation in Tudan Village, Sabah, a state in the region of East Malaysia, as the main mulberry plantation area [22]. The increasing plantation of mulberry in the urban or rural areas of various highlands and lowlands for both local and commercial consumption has certainly shown the important values of this plant. Nevertheless, this highland-cultivated mulberry is scarcely studied, hence their industrial underutilization. Reportedly, variation in cultivation location and condition as well as maturity stages, storage handling, processing technique, and parameters prompted different metabolites’ production in the plant [23]. These variations that consequentially influence the antioxidant activity of plants have been reported in studies with different environmental factors [23], different sample maturity levels [24], and different extraction solvents [22,25]. For example, despite analyzing the same mulberry species, Jelled et al. [15] obtained decreasing TPC, TFC, and antioxidant activities across fruits’ ripening stages, whereas Lee and Hwang [14] obtained increasing TPC, TFC, and antioxidant activities across fruits’ ripening stages. On the other hand, the effect of an extraction solvent is clearly demonstrated by the varying levels of TPC, TFC, and antioxidative ability of several vegetables extracted in 70% (*v*/*v*) methanol, 70% (*v*/*v*) ethanol, 70% (*v*/*v*) acetone, and distilled water [26]. Therefore, it is important to investigate the effect of maturity stages in fruits and leaves and to find the most efficient solvent for the extraction of mulberry’s bioactive compound and antioxidant activity. Methanol, ethanol, and acetone are among the organic solvents possessing excellent polarity widely used to extract natural compounds from plants [25,27]. Despite the reported toxicity of methanol, ethanol, and acetone [28,29], they have been widely used as solvents and reagents in organic chemistry [30]. By removing the residual solvents to the permissible level [31,32], methanol, ethanol, and acetone have been used in various industries [30], including the pharmaceutical industry, during the synthesis pathway of an active substance or excipients and the drug formulation process [33]. Therefore, in this study, local highland mulberry fruits and leaves at two different maturity stages (fruits: red mature (RF) and black fully ripe (BF); leaves: mature (ML) and young (YL)) and extracted in three different solvents (70% (*v*/*v*) methanol, 60% (*v*/*v*) ethanol, and 65% (*v*/*v*) acetone) were analyzed for their total bioactive compound content and antioxidant activity.

## 2. Results and Discussion

### 2.1. Extraction Yields

BFs showed a significant 20.93% higher extraction yield than RFs (Table 1). On the other hand, MLs showed a 7.95% higher extraction yield than YLs (Table 2). This result is owing to the rich content of biomolecules in the fruits and leaves of mulberry, such as macromolecules, polyphenolic, vitamins, and micromolecules [14]. The higher extraction yield of BFs can be associated with its higher level of protein, fatty acids, volatile compounds, and total carbohydrate [15]. On the other hand, the higher yield of MLs is possibly due to its greater amount of 1-deoxynojirimycin (DNJ), monoterpenes, and diterpenes as reported in Wulandari et al. [34].

For the assessment among solvents, 60% (*v*/*v*) ethanol extracts obtained the highest recovery yield in both fruit and leaf samples, which was 6.28% higher than that for 65% (*v*/*v*) acetone and 13.08% higher than that for 70% (*v*/*v*) methanol extracts. This result was similarly observed in Kobus-Cisowska et al. [25]. Accordingly, the extraction efficiency of plant phytochemicals depends on the polarity of solvents and chemical nature of the phytochemicals [35]. This means that the biomolecules in BFs and MLs are more soluble in 60% (*v*/*v*) ethanol and 65% (*v*/*v*) acetone, whereas the biomolecules in RFs and YLs are more soluble in 60% (*v*/*v*) ethanol and 70% (*v*/*v*) methanol. The higher recovery percentages in 60% (*v*/*v*) ethanol and 65% (*v*/*v*) acetone extracts might be related to their ability to dissolve phenols and endogenous compounds [25]. Nevertheless, these values are composed of both phenolic and nonphenolic substances in the sample, which are possibly attached to other biomolecules, such as lipids, chlorophyll, proteins, and organic or inorganic components [25,35]. Thus, an additional process and analysis are needed to remove the unwanted components.

### 2.2. Bioactive Compounds

#### 2.2.1. TPC of Mulberry Fruits and Leaves

Based on Table 1, the TPC values of BFs (2.32–6.91 mg GAE/g DW) are significantly higher than RFs (1.47–4.96 mg GAE/g DW) by 32.0% with 65% (*v*/*v*) acetone holding the highest values among the solvents. This maturity-dependent increment of TPC is similarly seen in another mulberry study with values of 1.1–3.2 g/100 g DW [14]. During fruit ripening, depolymerization of pectin, matrix glycans, and neutral sugars decrease the adhesion between the cells, leading to the softening of the cell wall tissue [36,37]. These effects, along with the shedding of cellulose and the distribution of a large amount of microfibrils in cell gaps, were reported in mulberry fruits across their ripening stages [38]. As a softer cell wall is stated to ease mastication and release of nutrients from the food matrix [39], the higher TPC values of BFs is believed to be owing to the changes of cell wall structural and mechanical properties upon ripening.

Meanwhile in Table 2, TPC decreases by 41.58% with the progression of leaf maturity. YLs obtained higher TPC values of 2.52–9.26 mg GAE/g DW compared with MLs (1.37–5.16 mg GAE/g DW) with 65% (*v*/*v*) acetone showing the highest TPC values. This result is supported by He et al. [40] with their higher TPC values in mulberry young leaves (27.35–30.03 mg GAE/g DW) than those in mature ones (16.30–17.26 mg QUE/g DW). The reduction of TPC across leaves’ maturity is the impact of degrading enzymes’ activation, which breaks phenolics down to some other secondary metabolites. This is confirmed via reabsorbance of degraded pigments in winter cherry leaves [24].

Among solvents, 65% (*v*/*v*) acetone extract showed the highest TPC values (4.96–9.26 mg GAE/g DW) in all samples and maturities, followed by 60% (*v*/*v*) ethanol (1.71–3.78 mg GAE/g DW) and 70% (*v*/*v*) methanol extracts (1.37–2.52 mg GAE/g DW). The TPC of 65% (*v*/*v*) acetone extracts, which was 58.96% higher than 60% (*v*/*v*) ethanol and 70.79% higher than 70% (*v*/*v*) methanol extracts, is in accordance with other fruit and vegetable studies [25,26]. The variation of values is attributable to the distinct polarity of compounds that significantly affects their extraction due to the “like–dissolve–like” selectivity of solvents [35]. These values are also due to the different response of Folin–Ciocalteu to the varying chemical structures of phenolics and antioxidant compounds [41]. Besides, the TPC results obtained from the three solvents in this study were higher compared with the 80% (*v*/*v*) ethanol and hot water (60 °C)-extracted mulberry fruits in our previous study [22]. The usage of 60% (*v*/*v*) ethanol in this study also exhibited better TPC compared with our previous 80% (*v*/*v*) ethanol-extracted mulberry (1.21 mg GAE/ mg DW) [22]. Hence, this indicated the compatibility and better phenolics extraction efficiency of 60% (*v*/*v*) ethanol to mulberry.

Nevertheless, an anomaly of lower TPC than TFC values was seen in this study. Accordingly, the reliability of the Folin–Ciocalteu method is affected by the matrix and hydroxyl group of the herbal matrix; the standard reference used and its molar absorption efficiency; temperature; pH; and concentration of chemicals in the chosen methodology [42,43,44]. For example, gallic acid is reported to give a better accuracy for phenolic estimation than other standard references (ferulic acid, chlorogenic acid, catechol, and vanillic acid) [44]. However, a comparison among other standard references reported the better sensitivity of pyrogallol and catechin in the Folin–Ciocalteu method compared with gallic acid [42,43]. These studies also reported different optimum conditions of the Folin–Ciocalteu method—standard reference, concentration of sodium carbonate (Na_2_CO_3_), analysis reaction time, and wavelength—in different types of plants. Therefore, the lower TPC in mulberry fruits and leaves might be due to the limitation of the extraction method and the Folin–Ciocalteu method employed, leading to TPC underquantification. A similar result was also demonstrated in other studies of mulberry fruits [45,46] and leaves [16,40].

#### 2.2.2. TFC of Mulberry Fruits and Leaves

Like TPC, BFs expressed 21.72% higher TFC values with 25.03–40.60 QUE/g DW compared with RFs (15.00–35.11 mg QUE/g DW), while 65% (*v*/*v*) acetone extracts exhibited the highest values in both maturities (Table 1). These data are supported by Lee and Hwang [14] with their increasing total flavonoid content across the maturation of mulberry fruits (0.1–0.4 g/100 g DW). Sharma et al. [47] believe that the higher TFC value in ripe fruits is owing to their more active production of phenolic compounds as a means of defense against stress and harm. Additionally, the sensory attribute profiling of mulberry fruits have demonstrated a positive relationship between their increased sweetness upon ripening and their increased secondary metabolites [48].

On the other hand, Table 2 reveals the significantly higher TFC values of YLs (23.00–45.32 mg QUE/g DW) than those of MLs (11.66–39.78 mg QUE/g DW). This 20.68% decrease in TFC with leaf maturity was also seen in He et al. [40] with 52.93–58.42 mg RE/g DW in young leaves and 27.61–28.78 mg RE/ g DW in mature leaves. Kumar et al. [49] mentioned that owing to the age and position of leaves on the plant, young leaves encompass a higher amount of nutrients, amino acids, and secondary metabolites than the mature leaves. Additionally, the lower TFC results of mature plants is because of the overaccumulation of reactive species, which reduces the radical scavenging activity of flavonoids [24].

In both fruits and leaves, 65% (*v*/*v*) acetone extracts obtained the greatest TFC values of 35.11–45.32 mg QUE/g DW, followed by 60% (*v*/*v*) ethanol (28.18–43.13 mg QUE/g DW) and 70% (*v*/*v*) methanol (11.66–25.03 mg QUE/g DW). The TFC of 65% (*v*/*v*) acetone was 11.28% higher than that of 60% (*v*/*v*) ethanol and 53.55% higher than that of 70% (*v*/*v*) methanol extracts. This result is supported by Tabart et al. [50], which reported acetone with water added as a better option in extracting protein matrices–polyphenols. This is because polyphenols tend to dissolve better in an organic solvent bearing lower polarity than water [51]. Acetone possesses the lowest polarity among the three utilized solvents, but its highest TFC values are conceivably owing to the increasing solvation efficiency related to the addition of water [52].

#### 2.2.3. TAC of Mulberry Fruits

The amount of TAC in BFs (2.34–7.15 mg Cya-3-Glu/g DW) was approximately 90.74% greater than that in RFs (0.16–0.77 mg Cya-3-Glu/g DW) (Table 1). This maturity-dependent increment is also shown in Lee and Hwang [14], which reported a dramatic rise of TAC from the immature to the fully mature mulberry fruits. This result is due to the increasing synthesis of anthocyanins throughout maturation that is associated with the color and sweet taste of fruits [53]. Changes of color in berries occur with the accumulation of carotenoids, anthocyanins, and betalains, as well as the loss of chlorophyll upon their ripening [53]. This explains the higher obtained TAC data of BFs as the brackish, fully ripe mulberry tastes sweeter than the reddish mature mulberry.

Contrary to TPC and TFC, 60% (*v*/*v*) ethanol extracts (0.77–7.15 mg Cya-3-Glu/g DW) presented the highest TAC values in both fruits’ maturity, followed by 70% (*v*/*v*) methanol (0.49–5.84 mg Cya-3-Glu/g DW) and 65% (*v*/*v*) acetone extracts (0.16–2.34 mg Cya-3-Glu/g DW). These results are remarkably higher than that of the TAC of 80% (*v*/*v*) ethanolic mulberry fruit extract (0.74 mg Cya-3-Glu/mg DW) in Centhyea et al. [22]. The higher TACs of 60% (*v*/*v*) ethanol and 70% (*v*/*v*) methanol than that of 65% (*v*/*v*) acetone (by 68.43% and 20.08%, respectively) are believed to be due to their ability to denature the cell membrane and dissolve and stabilize anthocyanins [35]. Meanwhile, the low TAC in 65% (*v*/*v*) acetone extracts is possibly due to its unusual response with anthocyanins, which produces pyranoanthocyanins. Pyranoanthocyanins are a group of more stable and complex polymeric anthocyanins that are not quantifiable via this assay [54]. In general, the TAC of mulberry fruits showed a maturity-dependent increment, and in both maturities, 60% (*v*/*v*) ethanol and 70% (*v*/*v*) methanol exhibited better efficiency in extracting anthocyanins.

#### 2.2.4. Quantification of Chlorogenic Acid and Rutin Using UHPLC-DAD

Chlorogenic acid (CGA) (C_16_H_18_O_9_) and rutin (C_27_H_30_O_16_) are two of the commonly found phenolic compounds in plants that possess various important biological activities, such as antioxidant, antiobesity, antihyperlipidemia, and anticarcinogenic [8,55,56]. These great benefits are the chief reason for the constant research in finding reliable sources as the presence and content of phenolic compounds vary depending on the type and part of the plant [14,49]. Despite the differing amounts, CGA and rutin are among the predominant phenolic compounds whose presence is confirmed in both the fruits and the leaves of mulberry [16,25,57,58]. Owing to their benefits and presence in mulberry, CGA and rutin have been quantified in some studies [16,40,58,59]. Hence, the detection and quantification of CGA and rutin were attempted in the fruits and leaves of this local highland mulberry.

**Figure 1:**
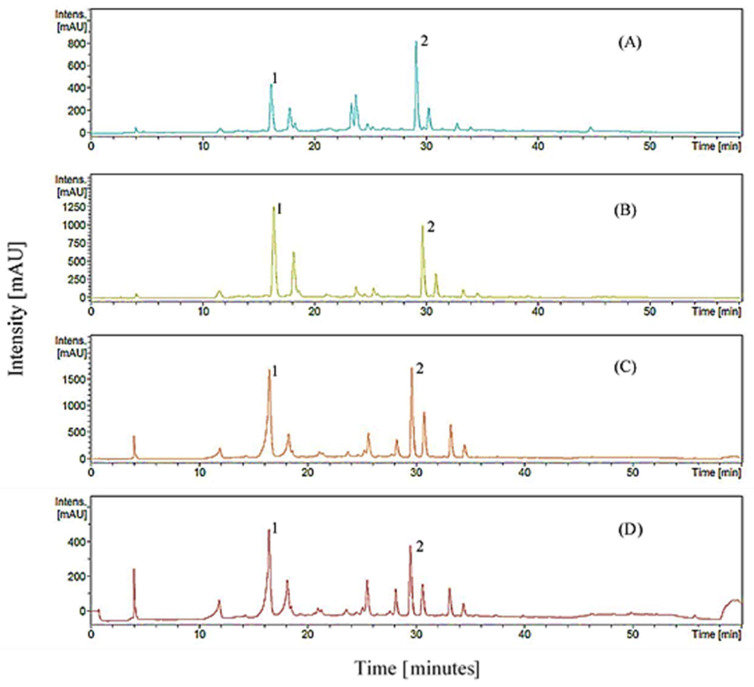
UHPLC chromatograms of mulberry fruits and leaves at different maturities and solvent extracts: (**A**) black fully ripe fruits in 65% (*v*/*v*) acetone; (**B**) red mature fruits in 65% (*v*/*v*) acetone; (**C**) young leaves in 60% (*v*/*v*) ethanol; (**D**) mature leaves in 60% (*v*/*v*) ethanol. 1, chlorogenic acid; 2, rutin.

Figure 1 shows the UHPLC-DAD spectra of CGA and rutin of different maturation mulberry fruits and leaves in different solvent extracts. Based on Table 1, the amounts of CGA and rutin in mulberry fruits decrease significantly across their ripening levels. For CGA, RFs obtained 60.75% significantly higher values (6.86–13.38 mg CGAE/g DW) than BFs (2.59–4.49 mg CGAE/g DW). On the other hand, for rutin, RFs obtained 2.58–4.93 mg RE/g DW, which was 8.88% greater than that of BFs (2.79–3.95 mg RE/g DW). These data are consistent with Lee and Hwang [14], where CGA and rutin values were significantly higher in the unripe mulberry fruits (3.92 mg/g DW and 664.6 mg/kg DW, respectively) than in the fully ripe fruits (0.58 mg/g DW and 592.0 mg/kg DW, respectively). CGA and rutin are biosynthesized through the phenylpropanoid pathway in which genes and enzymes are reported to downregulate across the ripening of fruits [60]. The transcriptions of CGA biosynthesis-related genes, phenylalanine ammonia lyase 1 (PAL1), caffeoyl-CoA 3-O-methyltransferase (CCoAMT), and 4-coumaroylester 3-Hydroxylase (C3′H) are remarkably lower in stage 4 coffee fruit and seed development as compared with the other stages [61]. On the other hand, the downregulation of rutin-related genes; PAL 2 and 3; chalcone synthase (CHS) 2, 3, and 9; cinnamate-4-hydroxylase (C4H) 2; and chalcone isomerase (CHI) 3 is also reduced across the mulberry fruit ripening process [60]. Hence, the higher values of CGA and rutin in RFs are possibly due to the downregulation of their genes in BFs as similarly seen in Zhao et al. [60].

For mulberry leaves (Table 2), CGA and rutin values declined with the leaves’ advancing maturity. YLs displayed 73.42% increased CGA values (8.93–30.73 mg CGAE/g DW) compared with MLs (1.30–8.78 mg CGAE/g DW). On the other hand, for rutin, YLs contained 3.56–8.70 mg RE/g DW of rutin, which was about 75.81% greater than that of MLs (0.83–2.26 mg RE/g DW). Similar data were reported by He et al. [40], in which mulberry young leaves obtained higher values of CGA and rutin (7.70–9.67 mg/g DW and 3.89–8.35 mg/g DW, respectively) compared with their mature leaves (4.10–8.72 mg/g DW and 1.09–4.41 mg/g DW, respectively). CGA and rutin are mostly located at the upper layer of leaves’ epidermis, and their photoprotective effect has been demonstrated in various plants [56,62]. Young leaves are more vulnerable to the deleterious effect of light and other stressors because of their thinner waxy surface and epidermal layers [63]. Young *C. arabica* leaves utilize CGA to counter the induced photodamage and the accumulated reactive oxygen species (ROS) [64]. Moreover, the sun-exposed young tartary buckwheat leaves reveal a remarkably higher activity of rutinosidase and rutin, which decrease with the aging of leaves [56]. Hence, the significantly higher CGA and rutin values of YLs are associated with their photoprotective function against the photodamage induced by strong sunlight.

The different extraction solvents show a significant variation of CGA and rutin values. In both ripenesses of fruits (Table 1), 65% (*v*/*v*) acetone extracts showed the highest CGA values (4.49–13.38 mg CGAE/g DW) with a difference of 27.53% and 47.12% from 60% (*v*/*v*) ethanol and 70% (*v*/*v*) methanol extracts, respectively. Meanwhile, 70% (*v*/*v*) methanol extracts held the utmost rutin values (3.95–4.93 mg RE/g DW), which were higher by 31.42% than those of 65% (*v*/*v*) acetone and by 35.92% than those of 60% (*v*/*v*) ethanol extracts. However, in both maturity levels of leaves (Table 2), 60% (*v*/*v*) ethanol extracts obtained the highest content of CGA (8.78–30.73 mg CGAE/g DW) with differences of 19.56% and 74.11% from those of 65% (*v*/*v*) acetone and 70% (*v*/*v*) methanol extracts, respectively. On the other hand, 65% (*v*/*v*) acetone exhibited the highest content of rutin (2.26–8.70 mg RE/g DW), which was greater by 5.38% than that of 60% (*v*/*v*) ethanol and by 59.95% than that of 70% (*v*/*v*) methanol extracts. Similar findings have been demonstrated in other mulberry studies [25,59,65]. CGA and rutin are compounds containing multiple hydroxyl groups that are highly soluble in alcohols and water [66,67]. The addition of water to the alcohol weakens the hydrogen bonds between the solvents and polyphenols and increases the basicity of the system and ionization of polyphenols [68]. In Hansen solubility parameters (HSPs), the closer is the distance between two molecules (R_a_) to zero (0), the better is their affinity [69,70]. For CGA, the R_a_ value increases in the order of methanol < ethanol < acetone [69]. Meanwhile, for rutin, the R_a_ value increases in the order of ethanol < methanol < acetone [70]. Nevertheless, the addition of water to these three solvents lowers their initial R_a_, which increases their affinity and solvation efficiency [69,70]. However, this increment varies depending on the ratio of water added. The 67% (*v*/*v*) acetone in Milescu et al. [70] showed the lowest R_a_ values of CGA and rutin compared with water mixed with ethanol and methanol solvents, thus supporting the high CGA and rutin values of 65% (*v*/*v*) acetone obtained in this study.

### 2.3. Antioxidant Analysis

#### 2.3.1. DPPH of Mulberry Fruits and Leaves

From Table 3, it is seen that the DPPH activity increases by 44.88% with the fruit maturity level. BFs exhibited lower IC_50_ = 0.073–0.152 mg/mL than RFs (IC_50_ = 0.16–0.77 mg/mL) with the 65% (*v*/*v*) acetone extract showing the lowest IC_50_ values in both maturities. This result is consistent with Lee and Hwang [14], which obtained increasing DPPH scavenging values from the unripe to fully ripe mulberry fruits (158–663 μmol/100 g DW). Meanwhile, Table 4 shows the 55.26% more potent DPPH scavenging activity of YLs based on its lower IC_50_ = 0.017–0.08 mg/mL than MLs (IC_50_ = 0.050–0.186 mg/mL) with 65% (*v*/*v*) acetone extracts showing the lowest IC_50_ value in both maturities. This result is similar to He et al. [40] where young mulberry leaves (27.94–30.90 mg/g) revealed higher DPPH results compared with the mature leaves (11.27–22.53 mg/g).

Previous studies have validated that mulberry fruits and leaves contain an abundant amount of antioxidative phenolic compounds that can act as a reductone against free radicals to inhibit radical chain reaction [19]. Therefore, the excellent activities of the fruits and leaves are owing to their high content of phenolic compounds as projected through their highly obtained TPC, TFC, and TAC values. Moreover, the decreasing DPPH activity in leaves is associated with the overproduction and accumulation of free radicals throughout plant growth, which depletes secondary metabolites and induces senescence in plant [24]. Xie and Schaich [71] reported that the DPPH scavenging ability of antioxidants is influenced by electron transfer (ET) or hydrogen atom transfer (HAT) action, which affects the speed of reaction, and by the structure complexity, the position, and the number of phenolic hydroxyl (-OH) groups that induce steric hindrance to the DPPH radical site. The potent DPPH scavenging compounds have been reported to be highly available in mulberry fruits and leaves, hence demonstrating the potent free radical scavenging activity of the fruits and leaves [19,40].

The 65% (*v*/*v*) acetone extracts (IC_50_ = 0.017–0.117 mg/mL) exhibited the highest DPPH potency in all fruits and leaves’ maturity levels. The result was then followed by 60% (*v*/*v*) ethanol (IC_50_ = 0.056–0.180 mg/mL) and 70% (*v*/*v*) methanol extracts (IC_50_ = 0.08–0.289 mg/mL). As such, the DPPH activity of 65% (*v*/*v*) acetone was higher by 41.59% than that of 60% (*v*/*v*) ethanol and by 63.65% than that of 70% (*v*/*v*) methanol. A similar result was seen in Arfan et al. [59] with the higher DPPH activity of the mulberry acetone extract (EC_50_ = 66 µg/mL) than that of the methanolic extract (EC_50_ = 79 µg/mL). Furthermore, this study demonstrated a higher DPPH activity compared with our previous 80% (*v*/*v*) ethanol and hot water (60 °C)-extracted mulberry fruits [22]. Besides, the 60% (*v*/*v*) ethanol extract presented a more potent DPPH activity compared with the 80% (*v*/*v*) ethanol extract [22], proving its closer polarity to antioxidants in mulberry. The variation of DPPH scavenging activity among the solvents is attributable to the effect of hydrogen bonding in polar solvents, which favors ET [72]. The hindered release of H atom will influence the action and rate of antioxidant activity towards DPPH radicals. Nevertheless, this assay limits the analysis of hydrophilic antioxidants due to the selective solubility of DPPH chromogens that dissolve only in organic solvents [73].

#### 2.3.2. ABTS of Mulberry Fruits and Leaves

Table 3 shows the 41.27% increment of ABTS values with fruits’ advancing maturity. BFs displayed higher ABTS values of 3.55–6.92 mg Tr/g DW compared with RFs (1.64–4.89 mg Tr/g DW) with the 65% (*v*/*v*) acetone extracts expressing the highest result in both fruits. The increasing ABTS values are consistent with Jelled et al. [15], which obtained an increasing ABTS value from the stage 1 mulberry fruits (2.58 mg/mL) to the stage 4 fruits (6.73 mg/mL). Meanwhile, Table 4 displays the 24.40% reduction of ABTS values across mulberry leaves’ progressing maturity. YLs exhibited higher ABTS values of 3.18–8.35 mg Tr/g DW compared with MLs (1.76–7.53 Tr/g DW) with the 65% (*v*/*v*) acetone extracts exhibiting the highest activity in both leaves’ samples. These decreasing ABTS values were similarly demonstrated by Thi and Hwang [74] with 35.9% ABTS scavenging activity in young aronia leaves and 23.4% in mature leaves.

These results are associated with the presence of potent ABTS scavenging compounds in mulberry, whose rate of activity depends on the structure of phenolics, their steric accessibility on the hindered ABTS radical site, and the formation of adducts [75,76]. Flavonoids such as quercetin, rutin, and morin are also potent ABTS scavengers due to their A, B, and C rings’ conjugated system, which stabilizes the formed radical [77]. Moreover, the presence of carbonyl and carboxyl groups in fruits and leaves greatly contributes to ABTS values [78]. The difference of activity in different maturity levels was in accordance with the amount of TPC, TFC, and TAC in each sample.

The 65% (*v*/*v*) acetone extracts obtained the highest ABTS values in all maturity levels of samples (4.89–8.35 mg Tr/g DW), which were 44.24% higher than the 60% (*v*/*v*) ethanol and 63.42% higher than 70% (*v*/*v*) methanol extracts. This result was similarly demonstrated by Arfan et al. [59], whose value was slightly higher in acetone extracted fruits (0.78 mmol Tr/g) than the methanolic extract (0.75 mmol Tr/g). This result is caused by the addition of water to polar solvent, which induces the stabilization of polar transition states essential for H atom abstraction [52,71]. Hence, the high ABTS values of the 65% (*v*/*v*) acetone extracts are possibly owing to the better facilitation of HAT compounds, which is enhanced by the addition of water. Unlike DPPH, ABTS allows the screening of both lipophilic and hydrophilic due to its solubility in both water and aqueous solvents. Nonetheless, the 6 minutes’ reaction time of ABTS was reported to be too short for the majority of antioxidants to achieve a steady state, especially the HAT antioxidants [79].

#### 2.3.3. FRAP of Mulberry Fruits and Leaves

Similar to the DPPH and ABTS assays, FRAP values increased with fruits’ progressing maturity with the 65% (*v*/*v*) acetone extracts holding the highest values. A 45.79% increment of FRAP values was seen between BFs (56.87–103.38 µM FeSO_4_/g DW) and RFs (37.14–58.86 µM FeSO_4_/g DW) (Table 3). This maturity-dependent increment of fruits is consistent with result obtained in Makavelou et al. [80]. In mulberry leaves, YLs expressed 55.51–195.49 µM FeSO_4_/g DW of FRAP values, which was 41.47% higher than that of MLs (24.87–91.86 µM FeSO_4_/g DW) with the 65% (*v*/*v*) acetone extracts having the highest values in both leaves (Table 4). Data are consistent with He et al. [40], which reported the lower FRAP values of mature mulberry leaves (18.67–29.49 mg/g) compared with young leaves (38.81–39.46 mg/g).

The presence of antioxidative compounds in mulberry fruits and leaves, including phenolics, flavonoids, tannins, organic acids, etc., are allegedly the key contributors of the samples’ reducing activity [25]. The extent of their antioxidant activity is influenced by the number and position of -OH groups, as well as the methylation of an -OH group [78,81]. Accordingly, the presence of two or more –OH phenolics in either ortho- or paraposition exhibits strong FRAP chelation. This property is owing to the delocalization of the conjugated aromatic ring system, which stabilizes the radicals and reduces an extra Fe^3+^-TPTZ complex [81]. Additionally, the methylation of an -OH group reduces the antioxidant efficiency due to the decreasing number of active electron- and hydrogen-donating groups [81]. The rich presence of these strong, medium, and weak reducing compounds has been validated in previous mulberry studies [19]. The variation of FRAP values in different maturities of samples was in accordance to the variation seen in their TPC, TFC, and TAC.

In a comparison of solvents, 65% (*v*/*v*) acetone (58.86–135.49 µM FeSO_4_/g DW) displayed the highest FRAP values in all sample maturity levels, followed by 60% (*v*/*v*) ethanol (40.81–94.27 µM FeSO_4_/g DW) and 70% (*v*/*v*) methanol (24.87–56.87 µM FeSO_4_/g DW) (Table 3 and Table 4). As such, the FRAP activity of 65% (*v*/*v*) acetone was 28.78% higher than that of 60% (*v*/*v*) ethanol and 55.24% higher than that of 70% (*v*/*v*) methanol extracts. A similar result is demonstrated in the leaves of various plants, including basil, mint, and aromatic ginger [26]. This effect of solvents is due to the influence of the antioxidants’ nature in the plant matrix: hydrophilic, lipophilic, entrapped in the cellular structure, and free or bound to macromolecules, which can be selectively and even partially soluble or insoluble in certain solvents [82]. Nevertheless, the nonspecificity of the FRAP assay allows any species with lower redox potential than that of Fe^3+^ (<0.70 V) to reduce Fe^3+^-TPTZ, leading to overestimation of the end values [73].

### 2.4. Principal Component Analysis (PCA)

The objective of conducting PCA in this study was to better describe the correlation and distribution of phenolic compounds and antioxidant activity in (1) mulberry fruits and (2) leaves of different maturities and in different solvent extractions. The Kaiser–Meyer–Olkin (KMO) test calculated a value of 0.6713 for our dataset; hence, it is suitable for PCA. The dataset exploration via PCA revealed PC1 and PC2 with an eigenvalue (EV) > 1, which explained the 81.381% cumulative variability (CV) of the dataset (Table 5). PC1 and PC2 provided information on variables with varying factor loading (FL) strengths as mentioned in Ismail et al. [83]. Strong FL ≥ |0.75| were TPC, TFC, DPPH, ABTS, and FRAP in PC1 and was TAC in PC2; moderate FL (|0.500| < FL < |0.749|) were CGA and rutin in PC1 and PC2; and weak FL ≤ |0.499| was TAC in PC1 and were TPC, TFC, DPPH, ABTS, and FRAP in PC2.

**Figure 2:**
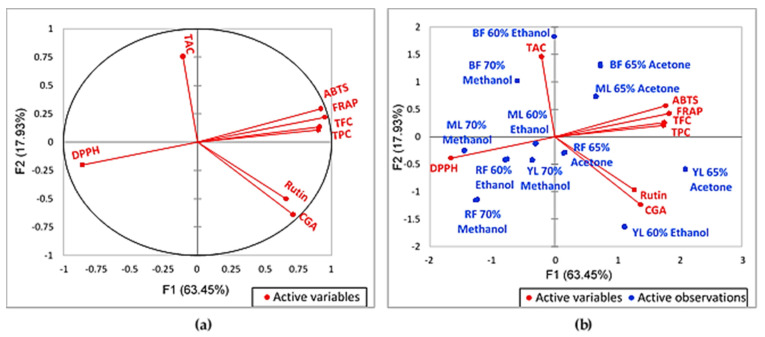
Plot of principal component analysis: (**a**) plot of total bioactive compound and antioxidant activity (axes F1 and F2: 81.38%); (**b**) correlation biplot of the active variables to mulberry fruits and leaf samples (axes F1 and F2: 81.23%). TPC, total phenolic content; TFC, total flavonoid content; TAC, total anthocyanin content; CGA, chlorogenic acid; BF, black fully ripe fruits; RF, red mature fruits; YL, young leaves; ML, mature leaves.

According to Figure 2a, this PCA explains a variance of 81.38%. A strong correlation was seen in TPC against TFC, ABTS, and FRAP, as well as in CGA against rutin, based on their close positions. On the other hand, a moderate correlation was seen in TPC, TFC, ABTS, and FRAP against CGA and rutin. The opposite located parameters indicate a negative correlation between them [84]. This negative correlation was seen in TAC against CGA and rutin, whose locations were almost in the opposite of one another. DPPH was also located on the opposite side of TPC, TFC, ABTS, and FRAP. This result supported the negative FL of DPPH in PC1 and PC2 (Table 5). Since DPPH was reported in IC_50_, whose lower IC_50_ values indicated higher antioxidant activity, this inverse correlation actually denoted their positive correlation. Hence, this means that DPPH antioxidant activity was strongly correlated to TPC, TFC, ABTS, and FRAP, while being moderately correlated to CGA and rutin. A weak correlation was seen in TAC against TPC, TFC, DPPH, ABTS, and FRAP. This result is not in line with other studies [85,86]. This is possibly because of the differing biochemical compositions and antioxidant activities of plants that are influenced by the genetic structure, environmental factors, and sample preparation and extraction methods [24,86]. Figure 2b demonstrates the biplots between eight variables (TPC, TFC, TAC, CGA, rutin, DPPH, FRAP, and ABTS) to their mulberry fruits and leaves of different maturities and solvent extracts (two maturities in three solvent extracts each).

Based on Figure 2b, BF 60% (*v*/*v*) ethanol and BF 70% (*v*/*v*) methanol contained the highest TAC based on their close distance. On the other hand, BF 65% (*v*/*v*) acetone contained the highest values for TPC, TFC, ABTS, and FRAP. Additionally, the opposite direction of BF 65% (*v*/*v*) acetone distance against DPPH indicated its stronger DPPH activity compared with BF 60% (*v*/*v*) ethanol and 70% (*v*/*v*) methanol extracts. However, the opposite direction of BF 60% (*v*/*v*) ethanol and 70% (*v*/*v*) methanol to CGA and rutin demonstrated their low content of these two compounds. This is consistent with the claim that rutin is used in the biosynthesis of anthocyanins across the fruit maturation process; hence, its content reduces along with the process [87]. On the other hand, RF 65% (*v*/*v*) acetone held a higher amount of CGA and rutin compared with all BF extracts because of its closer position to the two variables. Additionally, the further distance of RF 65% (*v*/*v*) acetone from DPPH implied its stronger antioxidant activity compared with RF 60% (*v*/*v*) ethanol and RF 70% (*v*/*v*) methanol. However, the middle position of RF 65% (*v*/*v*) acetone signified that all eight variables were low in content in this extract. Moreover, the close position of RF 60% (*v*/*v*) ethanol and 70% (*v*/*v*) methanol to DPPH as well as their far position from the other seven variables indicated their low TPC, TFC, and TAC content and antioxidant activities. The results of this biplot supported the prior obtained data tabulated in Table 1 and Table 3.

As regards mulberry leaf samples, the YL 65% (*v*/*v*) acetone and YL 60% (*v*/*v*) ethanol were closed to rutin and CGA, as well as farthest from DPPH compared with YL 70% (*v*/*v*) methanol. This demonstrated their higher CGA, rutin, and DPPH values than YL 70% (*v*/*v*) methanol. However, the closer position of YL 60% (*v*/*v*) ethanol to the CGA implied its higher CGA values compared with YL 65% (*v*/*v*) acetone. However, the YL 65% (*v*/*v*) acetone was positioned nearer to TPC, TFC, ABTS, and FRAP, which signified its high content of these variables and high antioxidant activity compared with YL 60% (*v*/*v*) ethanol, YL 70% (*v*/*v*) methanol, and the three ML extracts. In contrast, the ML 65% (*v*/*v*) acetone was positioned close to ABTS and FRAP, and at a farther position from DPPH, indicating its high possession of TPC, TFC, and stronger antioxidant activity compared with ML 60% (*v*/*v*) ethanol and 70% (*v*/*v*) methanol extracts. Besides, the closer distance of ML 60% (*v*/*v*) ethanol and ML 70% (*v*/*v*) methanol to DPPH and far distance to the other variables demonstrated their low level of TPC, TFC, CGA, rutin, and antioxidant activities. This biplot pattern (Figure 2b) supported the attained data shown in Table 2 and Table 4.

Overall, BF 65% (*v*/*v*) acetone is the best source for TPC, TFC, and antioxidant activity (DPPH, ABTS, and FRAP), but BF 60% (*v*/*v*) ethanol and 70% (*v*/*v*) methanol extracts are the better sources of TAC. Meanwhile, RF 65% (*v*/*v*) acetone is a more preferred source of CGA and rutin. As regards the leaf sample, YL 65% (*v*/*v*) acetone is the predominant source of TPC, TFC, rutin, and antioxidant activity (DPPH, ABTS, and FRAP), while YL 60% (*v*/*v*) ethanol is the best source of CGA. Despite their lower values than those of YL extracts, ML 65% (*v*/*v*) acetone contains higher TPC, TFC, CGA, rutin, and antioxidant activities (DPPH, ABTS, and FRAP) compared with the other ML extracts.

## 3. Materials and Methods

### 3.1. Sample Collection and Preparation

Mulberry fruits: maturity indexes 4 (red and mature) and 5 (black and fully ripe) [48] were harvested between September 2018 and 2019 and October 2018 and 2019 (harvesting time). Mulberry leaves: young leaves (the first to fourth leaves) and mature leaves (the fifth to eighth leaves) [34] were harvested between July and August 2020. The fruit and leaf samples were collected from the mulberry plantation in Tudan Village, Tuaran, Sabah, East Malaysia, as this is the main plantation area in Kota Kinabalu. The black fully ripe fruits (BF), red mature fruits (RF), young leaves (YL), and mature leaves (ML) were washed and left to dry for 10 min before being frozen overnight in an −80 °C freezer (New Brunswick Scientific U410, Hamburg, Germany). The samples were then freeze-dried (Labconco, Kansas city, MO, USA) at −40 °C with a pressure of 0.5 pa for 48 h and were respectively powdered using a laboratory blender (Waring 8010S, McConnellsburg, Pennsylvania, USA) at 18,000 rpm for 5 min.

### 3.2. Preparation of Mulberry Fruits and Leaf Extracts

Each of the powdered fruits and leaf samples was respectively extracted with 70% (*v*/*v*) methanol [58], 60% (*v*/*v*) ethanol [88], and 65% (*v*/*v*) acetone [89]. The extraction was conducted for 4 h in a shaking water bath (Daihan MaXturdy 30, Daihan Scientific Co., Ltd., Wonju, Korea) with 1:30 (m/v) at 60 °C. Extracts were then centrifuged (Eppendorf Centrifuge 5430R, Hamburg, Germany) at 26 °C and at 7745× *g* for 15 min before being filtered using Whatman No. 1 filter paper and rotary evaporated (Heidolph Laborota 4000, Brandenburg, Germany) at 30 °C to dry. Results were calculated with the formula and expressed as percentage recovery (%).

Percentage recover (%) = (mass of pure product recovered/mass of crude material used) × 100(1)

### 3.3. Determination of Phenolic Compounds

#### 3.3.1. Determination of Total Phenolic Content (TPC)

The TPC was carried out by using the Folin–Ciocalteu method based on Singleton and Rossi [90] with slight modifications. In this test, 1.5 mL of 1:10 (*v*/*v*) water-diluted Folin–Ciocalteu’s phenol reagent (Merck, EMD Millipore Corporation, Darmstadt, Germany) was added to 0.3 mL of the sample. After 10 min of incubation in the dark at room temperature, 1.2 mL of 7.5% (*w*/*v*) sodium carbonate (Merck, EMD Millipore Corporation, Darmstadt, Germany) solution was added. The mixture was then further incubated in the dark for 30 min. The absorbance was read at 743 nm using a UV–VIS spectrophotometer (Lambda 35 UV–VIS Spectrometer, PerkinElmer, Waltham, MA, USA), and gallic acid (Merck, EMD Millipore Corporation, Darmstadt, Germany) was used as a standard. The results were expressed as milligram of gallic acid equivalent per gram of dry weight (mg GAE/g DW).

#### 3.3.2. Determination of Total Flavonoid Content (TFC)

Aluminum chloride (AlCl_3_) assay according to Izzreen and Fadzelly [91] was utilized with modification. First, 0.5 mL of the sample, 2 mL of distilled water, and 0.15 mL of 10% (*w*/*v*) AlCl_3_ (Systerm, Classic Chemicals Sdn. Bhd., Shah Alam, Malaysia) solution were mixed. After 6 min of incubation in the dark at room temperature, 1 mL of 1 M sodium hydroxide (NaOH) (Sigma-Aldrich, Roche Diagnostics Deutschland GmbH, Burlington, MA, USA) and 1.2 mL of distilled water were added to the mixture. After 15 min of incubation in the dark at room temperature, the mixture was measured using a UV–VIS spectrophotometer (Lambda 35 UV–VIS Spectrometer, PerkinElmer, Waltham, MA, USA) at 510 nm. Quercetin (Sigma-Aldrich, Sigma-Aldrich Chemie GmbH, Bangalore, Karnataka, India) was used as a standard, and results were expressed as milligram of quercetin equivalent per gram of dry weight (mg QE/g DW).

#### 3.3.3. Determination of Total Anthocyanin Content (TAC)

This analysis utilized a pH difference spectrophotometric method as described by Giusti and Wrolstad [92] with minimal modification. First, two sets of 0.5 mL of the sample were respectively added with 3.5 mL of 0.025 M potassium chloride buffer (pH 1.0) (Systerm, Classic Chemicals Sdn. Bhd., Shah Alam, Malaysia) and 3.5 mL of 0.4 M sodium acetate buffer (pH 4.5) (Systerm, Classic Chemicals Sdn. Bhd., Shah Alam, Malaysia). Each mixture was analyzed at both 515 nm and 700 nm using a UV–VIS spectrophotometer (Lambda 35 UV–VIS Spectrometer, PerkinElmer, Waltham, MA, USA). Results were calculated with the formula and expressed as milligram of cyanidin-3-glucoside equivalent per gram of dry weight (mg C3GE/g DW):

TAC = (A × Mw × DF × 1000)/(*ε* × 1), (2)

where A is the absorption = (A515 − A700) of pH 1.0–(A515 − A700) of pH 4.5, Mw is the weight of cyanidin-3-glucoside molecule = 449.2, DF is the sample dilution factor, and *ε* is the molar absorption of cyanidin-3-glucoside = 26,900.

#### 3.3.4. Quantification of Chlorogenic Acid and Rutin Using UHPLC-DAD

The analysis was conducted using a Vanquish Flex Quaternary UHPLC system and diode array detector (DAD) (Thermo Fisher Scientific, Waltham, MA, USA) with Compass Hystar^TM^ 3.2 software (Bruker, Billerica, MA, USA). The analysis of compounds was performed with a 5 µL sample injection volume and a 0.5 mL/min flow rate pumped through a Phenyl Acclaim C18 reversed phase column (2.1 mm × 150 mm, 3 µm particle size, 120 A pore size) (Thermo Fisher scientific, Waltham, MA, USA) at 30 °C. The detection was performed online using a DAD detector at λ_260nm_–λ_360nm_.

Each sample was prefiltered with a 0.45 µm syringe filter before analysis and the two mobile phases: A = 99.9% water + 0.1% formic acid (H_2_O/CH_2_O_2_) and B = 99.9% acetonitrile + 0.1% formic acid (CH_3_CN/CH_2_O_2_) were sonicated for 15 min prior to usage. The UHPLC system was purged at 3 mL/min for 6 min prior to analysis. The column was washed at 0.5 mL/min for 10 min before and after analysis. The analysis was run for 60 min in gradient elution of 0–3 min: 5% B, 3–40 min: 5–40% B, 40–45 min: 25–35% B, 45–50 min: 35–50% B, 50–55 min: 50–45% B, 55.1–58 min: 45–95% B, 58–58.1 min: 95–5% B, 58.1–60 min: 5% B.

The identification of compounds was carried out via comparison of their retention times against CGA (Dr. Ehrenstorfer, LGC Limited, Teddington, England, United Kingdom) and rutin (Phyproof, PhytoLab GmbH & Co. KG, Bavaria, Germany) as external standards. The quantification was achieved by injection of known concentrations of CGA (0.02, 0.05, 0.1, 0.4, 0.6, and 1.0 mg/mL) and rutin (0.02, 0.05, 0.1, 0.4, 0.6, and 1.0 mg/mL).

### 3.4. Determination of Antioxidant Activity

#### 3.4.1. 2.2-Diphenyl-1-Picrylhydrazyl Radical Scavenging (DPPH) Assay

The assay was conducted according to Choi et al. [93] with minor alteration. First, 2.4 mL of 0.1 mM DPPH (Merck, EMD Millipore Corporation, Darmstadt, Germany) solution was added into 1.6 mL of the sample, vortexed (Heathrow Scientific, Vernon Hills, IL, USA), and incubated in the dark for 30 min at room temperature. The absorbance was measured at 517 nm using a UV–VIS spectrophotometer (Lambda 35 UV–VIS Spectrometer, PerkinElmer, Waltham, MA, USA). Trolox (Merck, EMD Millipore Corporation, Darmstadt, Germany) was used as a reference compound, and the percentages of DPPH radical scavenging activity was calculated by using the equation below. The results were reported as IC_50_:

DPPH (%) = [(A0 − A1)/A0] × 100,(3)

where A_0_ = abs of control (2.4 mL of 0.1 mM DPPH + 1.6 mL of 70% (*v*/*v*) methanol/60% (*v*/*v*) ethanol/65% (*v*/*v*) acetone, respectively) and A_1_ = abs of the sample (2.4 mL of 0.1 mM DPPH + 1.6 mL of the sample in respective solvents).

#### 3.4.2. 2,2′-Azino-Bis (3-Ethylbenzthiazoline-6-Sulphonic Acid) (ABTS) Assay

The ABTS assay was conducted based on Fu et al. [94] with modification. The working solution consisted of 5 mL of 7 mM ABTS (Sigma-Aldrich, Roche Diagnostics Deutschland GmbH, Burlington, MA, USA) solution and 5 mL of 2.45 mM potassium persulfate (K_2_S_2_O_8_) (Systerm, Classic Chemicals Sdn. Bhd., Shah Alam, Malaysia) solution, first prepared and kept in the dark for 16 h at room temperature. Before usage, the ABTS solution was diluted with water to a reading of 0.70 ± 0.05 at 734 nm. Then, 3.80 mL of the diluted ABTS was added to 100 µL of the sample and kept in the dark for 6 min at room temperature before being analyzed using a UV–VIS spectrophotometer (Lambda 35 UV–VIS Spectrometer, PerkinElmer, Waltham, MA, USA) at 734 nm. Trolox (Merck, EMD Millipore Corporation, Darmstadt, Germany) was used as a standard, and the results were expressed as milligram of trolox equivalents per gram of dry weight (mg TE/g DW).

#### 3.4.3. Ferric-Reducing Antioxidant Power (FRAP) Assay

FRAP assay was carried out according to Fu et al. [94] with minimal modification. First, 300 mM acetate buffer, 10 mM 2,4,6-Tris(2-pyridyl)-1,3,5-triazine (TPTZ) solution (Sigma-Aldrich, Roche Diagnostics Deutschland GmbH, Burlington, MA, USA), and 20 mM iron (III) chloride hexahydrate (FeCl_3_·6H_2_O) solution (Merck, EMD Millipore Corporation, Darmstadt, Germany) were added successively at a ratio of 10:1:1 (v/v/v). The working solution was incubated at 37 °C in water bath (Daihan MaXturdy 30, Daihan Scientific Co., Ltd., Wonju, Korea) before usage. Next, 3 mL of working solution was added to 100 µL of the sample and incubated for 4 min before a UV–VIS spectrophotometer (Lambda 35 UV–VIS Spectrometer, PerkinElmer, Waltham, MA, USA) reading at 593 nm. Iron (II) sulfate heptahydrate (FeSO_4_.7H_2_O) (Systerm, Classic Chemicals Sdn. Bhd., Shah Alam, Selangor, Malaysia) was used as a standard for a calibration curve, and results were expressed as the concentration of antioxidants having the ability to reduce ferric per gram of dry weight (µM FeSO_4_/g DW).

### 3.5. Analysis of Data

All experiments were performed in triplicate. All data are expressed as the mean ± standard deviation. To determine the effect of two independent factors (maturation stages and extraction solvents) on the dependent factors (phenolic content and antioxidant activity of mulberry), basic descriptive statistical analysis was performed among the means using two-way analysis of variance (ANOVA) (IBM SPSS Statistics version 23.0, IBM Corp., Armonk, NY, USA). When two-way ANOVA showed a significant difference, one-way ANOVA and Tukey’s HSD comparison test at a 95% significance level (*p* < 0.05) were conducted to see the significant difference among samples (IBM SPSS Statistics version 23.0, IBM Corp., Armonk, NY, USA). The Kaiser–Meyer–Olkin (KMO) test and principal component analysis (PCA) were conducted using XLSTAT-Pro (version 2017.1) statistical software (Addinsoft, Paris, France).

## 4. Conclusions

In conclusion, the highland-cultivated mulberry exhibits high quantity of bioactive compounds and antioxidant activity, which are significantly influenced by maturity stages and extraction solvents. Mulberry fruits demonstrate a maturity-dependent increment as the black fully ripe mulberry fruits reveal a higher total bioactive compound content and antioxidant activity than the red mature fruits. However, red mature fruits contain a higher amount of chlorogenic acid and rutin instead. On the other hand, mulberry leaves demonstrate maturity-dependent reduction based on the higher total bioactive compound content, chlorogenic acid, rutin, and antioxidant activity of young leaves than mature leaves. Additionally, in all analyses (excluding total anthocyanin content and UHPLC-DAD quantification of chlorogenic acid in leaves and rutin in fruits), the 65% (*v*/*v*) acetone extracts of the fruits and leaves possess significantly higher values than 60% (*v*/*v*) ethanol and 70% (*v*/*v*) methanol extracts, indicating the efficiency and compatibility of 65% (*v*/*v*) acetone with mulberry. For mulberry fruits, the biplot of PCA shows black fully ripe fruits in 65% (*v*/*v*) acetone extract as the best phenolic, flavonoid, and antioxidant source. However, the black fully ripe fruits in 60% (*v*/*v*) ethanol and 70% (*v*/*v*) methanol are better anthocyanin sources. Meanwhile, the red mature fruits in 65% (*v*/*v*) acetone is a better source of chlorogenic acid, and its 70% (*v*/*v*) methanol is a better source of rutin. On the other hand, for mulberry leaves, a PCA biplot displays young leaves in 65% (*v*/*v*) acetone as the best phenolic, flavonoid, rutin, and antioxidant sources. The young leaves in 60% (*v*/*v*) ethanol is a better source of chlorogenic acid than other leaf extracts. Overall, the fruits and leaves of mulberry contain a rich amount of phenolics and strong antioxidant capacity to be utilized in food and pharmaceutical products. Nevertheless, the compound quantification, bioavailability, and in vitro and in vivo antioxidant activity ought to be focused on future study, as more comprehensive data on highland-grown mulberry are needed prior to their product development.

## Figures and Tables

**Table 1:** The values of extraction yield, total bioactive content, chlorogenic acid, and rutin of mulberry fruits. The result of two-way ANOVA are shown for the interaction of the two factors: maturation stages (ms) and extraction solvent (es).

Analysis	Result in Different Maturities and Different Solvents						Two-Way ANOVA
	BF			RF			ms*es (*p*-Value)
	MeOH	EtOH	Acetone	MeOH	EtOH	Acetone	
Extraction Yield (%)	52.53 ± 0.95 ^b^	68.21 ± 0.65 ^a^	67.13 ± 0.41 ^a^	49.71 ± 0.10 ^c^	52.33 ± 0.59 ^b^	46.50 ± 0.24 ^d^	<0.001
TPC (mg GAE/g DW)	2.32 ± 0.01 ^d^	2.74 ± 0.01 ^c^	6.91 ± 0.01 ^a^	1.47 ± 0.00 ^e^	1.71 ± 0.01 ^f^	4.96 ± 0.01 ^b^	<0.001
TFC (mg QUE/g DW)	25.03 ± 0.05 ^e^	34.38 ± 0.04 ^c^	40.60 ± 0.07 ^a^	15.00 ± 0.04 ^f^	28.18 ± 0.05 ^d^	35.11 ± 0.04 ^b^	<0.001
TAC (mg Cya-3-Glu/g DW)	5.84 ± 0.01 ^b^	7.15 ± 0.01 ^a^	2.34 ± 0.14 ^c^	0.49 ± 0.01 ^e^	0.77 ± 0.02 ^d^	0.16 ± 0.01 ^f^	<0.001
CGA(mg CGAE/g DW)	2.59 ± 0.01 ^f^	4.27 ± 0.03 ^e^	4.49 ± 0.01 ^d^	6.86 ± 0.02 ^c^	8.68± 0.00 ^b^	13.38 ± 0.02 ^a^	<0.001
Rutin (mg RE/g DW)	3.95 ± 0.01 ^b^	3.11 ± 0.01 ^d^	2.79 ± 0.02 ^e^	4.93 ± 0.03 ^a^	2.58 ± 0.01 ^f^	3.30 ± 0.02 ^c^	<0.001

Data are expressed as mean ± SD (*n* = 6). Different superscript letters in the same row indicate significant difference (*p* < 0.05). BF, black fully ripe fruits; RF, red mature fruits; MeOH, 70% (*v*/*v*) methanol; EtOH, 60% (*v*/*v*) ethanol; acetone, 65% (*v*/*v*) acetone; TPC, total phenolic content; TFC, total flavonoid content; TAC, total anthocyanin content; CGA, chlorogenic acid. If a significant interaction effect was found in ms*es, one-way ANOVA on the combination factor of both effects was run. The interaction effect is the most important effect.

**Table 2:** The values of extraction yield, total bioactive content, chlorogenic acid, and rutin of mulberry leaves. The result of two-way ANOVA is shown for the interaction of the two factors: maturation stages (ms) and extraction solvent (es).

Analysis	Result in Different Maturities and Different Solvents						Two-Way ANOVA
	YL			ML			ms*es (*p*-Value)
	MeOH	EtOH	Acetone	MeOH	EtOH	Acetone	
Extraction Yield (%)	24.07 ± 0.79	26.07 ± 0.71	23.00 ± 0.81	25.14 ± 0.39	27.64 ± 0.51	26.68 ± 0.32	>0.05
TPC (mg GAE/g DW)	2.52 ± 0.01 ^e^	3.78 ± 0.01 ^c^	9.26 ± 0.01 ^a^	1.37 ± 0.00 ^f^	2.56 ± 0.01 ^d^	5.16 ± 0.01 ^b^	<0.001
TFC (mg QUE/g DW)	23.00 ± 0.04 ^e^	43.13 ± 0.03 ^b^	45.32 ± 0.07 ^a^	11.66 ± 0.05 ^f^	36.97 ± 0.05 ^d^	39.77 ± 0.05 ^c^	<0.001
CGA (mg CGAE/g DW)	8.93 ± 0.02 ^c^	30.73 ± 0.10 ^a^	24.74 ± 0.02 ^b^	1.30 ± 0.01 ^f^	8.78 ± 0.05 ^d^	7.04 ± 0.03 ^e^	<0.001
Rutin (mg RE/g DW)	3.56 ± 0.01 ^c^	8.45 ± 0.03 ^b^	8.70 ± 0.02 ^a^	0.83 ± 0.00 ^f^	1.92 ± 0.00 ^e^	2.26 ± 0.01 ^d^	<0.001

Data are expressed as mean ± SD (*n* = 6). Different superscript letters in the same row indicates significant difference (*p* < 0.05). YL, Young leaves; ML, Mature leaves; MeOH, 70% (*v*/*v*) Methanol; EtOH, 60% (*v*/*v*) Ethanol; Acetone, 65% (*v*/*v*) Acetone; TPC, Total phenolic content; TFC, Total flavonoid content; CGA, Chlorogenic acid. If a significant interaction effect was found between ms*es, one-way ANOVA on the combination factor of both effects was run. The interaction effect is the most important effect.

**Table 3:** The antioxidant activity of mulberry fruits. The results of two-way ANOVA are shown for the interaction of the two factors: maturation stages (ms) and extraction solvent (es).

Analysis	Result in Different Maturities and Different Solvents						Two-Way ANOVA
	BF			RF			ms*es (*p*-Value)
	MeOH	EtOH	Acetone	MeOH	EtOH	Acetone	
DPPH (IC_50_)	0.152 ± 0.00 ^c^	0.098 ± 0.00 ^d^	0.073 ± 0.00 ^e^	0.289 ± 0.01 ^a^	0.180 ± 0.01 ^b^	0.117 ± 0.01 ^d^	<0.001
ABTS (mg Tr/g DW)	3.55 ± 0.01 ^d^	4.82 ± 0.01 ^c^	6.92 ± 0.01 ^a^	1.64 ± 0.00 ^f^	2.45 ± 0.01 ^e^	4.89 ± 0.01 ^b^	<0.001
FRAP (µM FeSO_4_/g DW)	56.87 ± 0.04 ^d^	92.12 ± 0.12 ^b^	103.38 ± 0.19 ^a^	37.14 ± 0.05 ^f^	40.81 ± 0.05 ^e^	58.86 ± 0.09 ^c^	<0.001

Data are expressed as mean ± SD (*n* = 6). Different superscript letters in the same row indicate significant difference (*p* < 0.05). BF, black fully ripe fruits; RF, red mature fruits; MeOH, 70% (*v*/*v*) methanol; EtOH, 60% (*v*/*v*) ethanol; acetone, 65% (*v*/*v*) acetone. If a significant interaction effect was found in ms*es, one-way ANOVA on the combination factor of both effects was run. The interaction effect is the most important effect.

**Table 4:** The antioxidant activity of mulberry leaves. The results of two-way ANOVA are shown for the interaction of the two factors: maturation stages (ms) and extraction solvent (es).

Analysis	Result in Different Maturities and Different Solvents						Two-Way ANOVA
	YL			ML			ms*es (*p*-Value)
	MeOH	EtOH	Acetone	MeOH	EtOH	Acetone	
DPPH (IC_50_)	0.080 ± 0.00 ^d^	0.056 ± 0.00 ^c^	0.017 ± 0.00 ^e^	0.186 ± 0.00 ^a^	0.106 ± 0.00 ^b^	0.050 ± 0.00 ^c^	<0.001
ABTS (mg Tr/g DW)	3.18 ± 0.01 ^d^	4.98 ± 0.01 ^c^	8.35 ± 0.01 ^a^	1.76 ± 0.01 ^e^	3.19 ± 0.01 ^d^	7.53 ± 0.01 ^b^	<0.001
FRAP (µM FeSO_4_/g DW)	55.51 ± 0.04 ^d^	94.27± 0.12 ^b^	135.49 ± 0.22 ^a^	24.87 ± 0.06 ^f^	50.25 ± 0.08 ^e^	91.86 ± 0.15 ^c^	<0.001

Data are expressed as mean ± SD (*n* = 6). Different superscript letters in the same row indicate significant difference (*p* < 0.05). YL, young leaves; ML, mature leaves; MeOH, 70% (*v*/*v*) methanol; EtOH, 60% (*v*/*v*) ethanol; acetone, 65% (*v*/*v*) acetone. If a significant interaction effect was found in ms*es, one-way ANOVA on the combination factor of both effects was run. The interaction effect is the most important effect.

**Table 5:** Eigenvalues, variability, cumulative variability, and factor loadings associated with each principal component.

Variables		PC1	PC2
Eigenvalues		5.0759	1.4346
Variability (%)		63.4483	17.9330
Cumulative variability (%)		64.4483	81.3813
Factor loadings	TPC	0.9000	0.1072
	TFC	0.9087	0.1339
	TAC	−0.1090	0.7553
	CGA	0.7124	−0.6401
	Rutin	0.6602	−0.5004
	DPPH	−0.8600	−0.2012
	ABTS	0.9194	0.2934
	FRAP	0.9486	0.2190

TPC, total phenolic content; TFC, total flavonoid content; TAC, total anthocyanin content; CGA, chlorogenic acid.

## Data Availability

Reported data-supporting results can be found in any masthead.

## Sample Availability

The samples’ availability is as stated in Section 3, Material and methods.
